# Safety of Seasonal Malaria Chemoprevention (SMC) with Sulfadoxine-Pyrimethamine plus Amodiaquine when Delivered to Children under 10 Years of Age by District Health Services in Senegal: Results from a Stepped-Wedge Cluster Randomized Trial

**DOI:** 10.1371/journal.pone.0162563

**Published:** 2016-10-20

**Authors:** J. L. NDiaye, B. Cissé, E. H. Ba, J. F. Gomis, C. T. Ndour, J. F. Molez, F. B. Fall, C. Sokhna, B. Faye, E. Kouevijdin, F. K. Niane, M. Cairns, J. F. Trape, C. Rogier, O. Gaye, B. M. Greenwood, P. J. M. Milligan

**Affiliations:** 1 Department of Parasitology, University Cheikh Anta Diop, Dakar, Senegal; 2 Institut de Recherche pour le Développement, Dakar, Sénégal; 3 National Malaria Control Program, Ministry of Health and Prevention, Dakar, Senegal; 4 Institut Pasteur, Antananarivo, Madagascar; 5 London School of Hygiene &Tropical Medicine, London, United Kingdom; University of Tübingen, GERMANY

## Abstract

**Background:**

It is recommended that children aged 3 months to five years of age living in areas of seasonal transmission in the sub-Sahel should receive Seasonal Malaria Chemoprevention (SMC) with sulfadoxine-pyrimethamine plus amodiaquine (SPAQ) during the malaria transmission season. The purpose of this study was to evaluate the safety of SMC with SPAQ in children when delivered by community health workers in three districts in Senegal where SMC was introduced over three years, in children from 3 months of age to five years of age in the first year, then in children up to 10 years of age.

**Methods:**

A surveillance system was established to record all deaths and all malaria cases diagnosed at health facilities and a pharmacovigilance system was established to detect adverse drug reactions. Health posts were randomized to introduce SMC in a stepped wedge design. SMC with SPAQ was administered once per month from September to November, by nine health-posts in 2008, by 27 in 2009 and by 45 in 2010.

**Results:**

After three years, 780,000 documented courses of SMC had been administered. High coverage was achieved. No serious adverse events attributable to the intervention were detected, despite a high level of surveillance.

**Conclusions:**

SMC is being implemented in countries of the sub-Sahel for children under 5 years of age, but in some areas the age distribution of cases of malaria may justify extending this age limit, as has been done in Senegal. Our results show that SMC is well tolerated in children under five and in older children. However, pharmacovigilance should be maintained where SMC is implemented and provision for strengthening national pharmacovigilance systems should be included in plans for SMC implementation.

**Trial Registration:**

ClinicalTrials.gov NCT 00712374

## Introduction

Seasonal Malaria Chemoprevention (SMC) is the administration of a therapeutic dose of antimalarials at monthly intervals to all children, regardless of whether they have malaria infection, to protect them from clinical attacks of malaria during the period of the year when malaria risk is greatest. SMC with sulphadoxine-pyrimethamine and amodiaquine (SPAQ) provides a high degree of protection against severe and uncomplicated malaria and is well tolerated when given in the context of clinical trials [1]. As a consequence, the World Health Organisation (WHO) recommended in March 2012 that children who live in areas of highly seasonal malaria transmission in the Sahel and sub-Sahel regions should receive SMC with SPAQ administered monthly for up to four months of the year during the period when children are at greatest risk of malaria [2,3]. An implementation guide was produced in November of the same year [4] and National Malaria Control Programmes have been quick to adopt this strategy. Twelve countries have now included SMC in their strategic plans for malaria control and implementation has started in ten countries, Burkina Faso, Chad, The Gambia, Ghana, Guinea, Mali, Niger, Nigeria and Togo for children under five years of age and in southern Senegal for children under 10 years of age. The aim of the study reported in this paper, conducted from 2008 to 2011, was to evaluate the safety of SMC with SPAQ when delivered on a large scale by district health services using community health workers (CHWs), as part of large study to evaluate the effectiveness of SMC.

## Materials and Methods

### Study population

**Fig 1:**
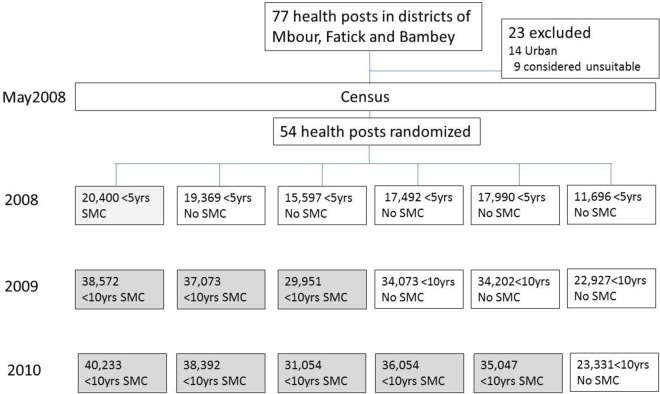
Trial profile, showing the number of children in each zone in the stepped-wedge trial.

**Fig 2:**
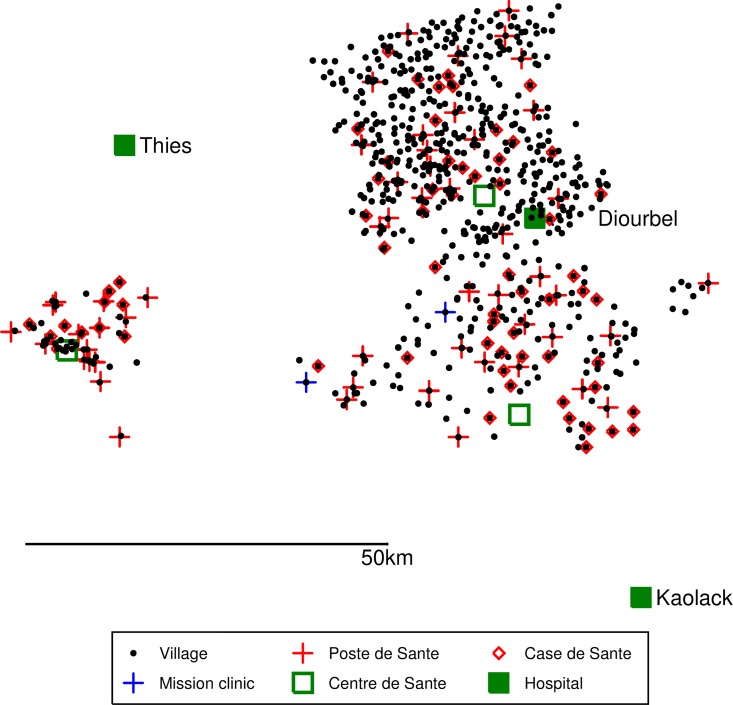
Map of the study area showing health facilities involved in SMC delivery and pharmacovigilance.

The study was conducted in three districts (Mbour, Bambey and Fatick) of Senegal (Figs 1 and 2). The population of the study area was about 600,000 in May 2008, of whom approximately 108,000 were aged 0–59 months and 98,000 aged 60–120 months. The population is served by 54 health posts and 72 “cases de santé” (health huts). In addition, there are 15 health centres in districts adjacent to the study area which may be used occasionally by people from the study area, and there are three referral hospitals (in Kaolack, Diourbel and Thies) and three district health centres in the study area. From 2008, the health posts and the majority of the “cases de santé” provided malaria diagnosis using Rapid Diagnostic Tests (RDT’s) and treatment with artemisinin combination therapy if the test was positive. Uncomplicated malaria was treated with amodiaquine-artesunate in 2008 and from 2009 with artemether-lumefantrine. From September 2007, a clinical algorithm was introduced into all health posts and health centres for the diagnosis and treatment of patients presenting with a febrile illness; this requires children to be tested with a RDT when there is a fever with no obvious non-malarial cause. An antibiotic (usually amoxicillin) is prescribed for cases of fever when the RDT is negative. Children under five years receive vitamin A and anthelmintics twice each year, and in one district (Bambey), mass treatment with azithromycin was provided during the study period. A national pharmacovigilance system, initiated in 1998, was extended in 2007 to assess adverse reactions to antimalarial drugs. However, prior to our study there was no systematic reporting of adverse events in the study area. The prevalence of HIV is low, the 2010–11 DHS survey in the general adult (15–45yrs) population found prevalence of HIV1 of 0.5% and HIV2 0.2% [5].

### Study design

Fifty-four health posts serving rural and semi-urban populations were randomized to implement SMC for children in either 2008, 2009, or 2010, with nine health posts remaining without the intervention. Details of the study design, sample size, and randomization process, are described in [6]. The intervention comprised a therapeutic dose of AQ (10 mg/kg/day for 3 days) combined with one dose of SP on the first day (25mg sulfamethoxypirazyne and 1.25mg pyrimethamine per kg in 2008, 25mg sulfadoxine, 1.25mg pyrimethamine in 2009–10) administered once per month for the last three months of the malaria transmission season (September-November). In 2008, SMC was given by district staff in nine health posts to children aged 3–59 months at the time of the first round of treatment. In 2009 and 2010, SMC was implemented in 27 health posts and 45 health posts respectively, and children aged 3 to 120 months were included. A surveillance system was established in 2008, before the start of the transmission period, to record all deaths among children under 10 years of age, to document all malaria cases diagnosed at health facilities, and to detect adverse drug reactions and adverse events that might be drug related. After a census was conducted in May 2008, households were visited once every 10 months to record births, deaths and changes in occupancy. In twelve health posts, purposively selected to be representative of the study area, all deaths under 10 years of age in the catchment population were investigated by verbal autopsy. A village reporter was recruited in each village who recorded all deaths of children under 10 years of age, and verbal autopsies were done using a modified version of the INDEPTH questionnaire (http://www.indepthnetwork.org/index.php?option=com_content&task=view&id=96&Itemid=184). Completed forms were reviewed by two physicians who ascribed a cause of death, forms were then reviewed by a third physician, and if there was any disagreement cause of death was agreed by a panel.

At the end of the 2008, 2009 and 2010 transmission seasons, a cross-sectional survey was conducted to determine coverage with SMC and reasons for missed doses, to record bednet use by children after inspecting the place where the child slept, to measure the prevalence of parasitaemia and anaemia, and to ask about any adverse events related to SMC.

The primary endpoints of the study were all cause mortality, malaria cases at outpatient clinics, and the incidence of adverse events. The results for mortality and malaria incidence are described elsewhere [6].

### Drug dosage and administration

Source of drugs and dosage are shown in Table 1. Tablets were tested and passed standard criteria for drug content, uniformity of content, dissolution and impurities at the Laboratoire National de Contrôle des Médicaments, Dakar. Dosage of SPAQ based on age was calculated from analysis of anthropometric survey data (K Simondon unpublished data 2004) in order to minimize under- and over-dosing while keeping simple dosage recommendations and avoiding use of quarter tablets.

**Table 1:** Source of drugs, and dosage by age, in each year.

	Drugs used	Dosage
2008	200mg amodiaquine tablets (Pfizer, Dakar)	AQ 200mg: 0.5 tablet (<2yrs); 1 tablet (2–4 yrs)
	sulfamethoxypirazyne (sulfalene) 500mg/ pyrimethamine 25mg (Pfizer, Dakar)	SP 500mg tablets: 0.5 tablet (<2yrs); 1 tablet (2–4 yrs)
2009	200mg amodiaquine tablets (Chongqing Qinyang Pharmaceutical Co Ltd)	AQ 200mg: 0.5 tablet (<2yrs); 1 tablet (2–8 yrs); 1.5 tablets (9–10yrs)
	SP (500mg sulfadoxine/25mg pyrimethamine, Shijizhuang Ouyi Pharmaceutical Co Ltd)	SP 500mg tablets: 0.5 tablet (<2yrs); 1 tablet (2–5 yrs); 1.5 tablets (6–10yrs)
2010	153mg amodiaquine, dispersible sweetened tablets (153mg, Kinapharma, Ghana)	AQ 153mg: 0.5tablet (<2yrs); 1 tablet (2–5 yrs); 1.5 tablets (6–10yrs)
	dispersible SP tablets (500mg sulfadoxine/25mg pyrimethamine, Kinapharma Ghana)	SP 500mg tablets: 0.5 tablet (<2yrs); 1 tablet (2–5 yrs); 1.5 tablets (6–10yrs)

### Surveillance for adverse events

At the household visit to deliver the first monthly round of SMC, CHWs explained to the mother/carer the purpose of the intervention and, in simple terms, the potential risks (that all drugs can cause side effects in some children, that AQ can cause vomiting and that SP can sometimes cause skin reactions, but that severe problems are rare with these drugs). The mothers/carers were instructed that if side effects of SMC treatment were suspected, the child should be brought to a health post without delay. CHWs visited each child one month after the first and second round of treatment to check that there had been no severe reactions to the previous treatment, and to give the next round of treatment. Training workshops were held for health post nurses and hospital staff to explain how to recognize and manage adverse drug reactions and how to document and report any adverse events suspected to be drug related. Severe skin reactions and signs of liver disease, and severe vomiting, were highlighted as adverse events of special interest, and the importance of laboratory investigation (blood count for detection of agranulocytosis, and liver function tests) was emphasised. Health staff in all facilities (health posts delivering SMC and health posts not delivering SMC) were asked to document and report any such cases regardless of a suspected relationship with drug intake. A leaflet with photographs and descriptions illustrating the most common features of adverse drug reactions to SP or AQ was prepared and used in training sessions (S1 Fig). Copies were given to all health posts and health centres in the study area. A reminder system was implemented using text messages; 60 health staff (54 nurses in the health posts and the key staff member at each of the 6 hospitals/health centres) were sent regular SMS messages reminding them to look out for adverse events, asking them to report by SMS any serious events. A phone credit was sent to them as an incentive, and they were contacted after SMC rounds to confirm whether any adverse events had been seen. Nurses were linked to a member of the project staff who contacted them by phone or in person during the study to maintain contact, ask about any problems, give support and advice, and remind them about study procedures using a standardized list of reminders. During the periods when SMC was being delivered, a supervisory visit was made to each health post each month by either the district medical officer or one of the project field supervisors. Each district supervisor had a digital camera to be used to document any skin rash.

Three hospitals and three health centres with inpatient facilities were involved in surveillance for signs of drug-related serious adverse events among children who were hospitalized. An additional 12 health facilities, adjacent to the study area, which were less likely to receive patients from the study area, also participated in the surveillance process; staff of these hospitals were informed about the project and asked to report any admissions of children from the study area.

After each transmission period, all inpatient records from the three health centres and three hospitals were collected and computerised for retrospective analysis to look for any adverse events that might have been drug-related but not reported. Deaths investigated by verbal autopsy (in 2 health posts where SMC was delivered in 2008, 6 health posts in 2009 and 10 health post in 2010) were reviewed for possible association with SMC drugs. In 2009 and 2010, documentation of adverse events at health posts was improved by training and supervising health staff to report all suspected adverse drug reactions using the national pharmacovigilance form. Hospital surveillance was improved by introducing a form for collecting inpatient information, patient identification, clinical signs and symptoms, laboratory results, diagnosis at admission and discharge, and details of medication taken in the previous two weeks in a standardized way. Workshops were held at which clinical staff from the six hospitals developed the inpatient record form with project staff, and produced guidelines for recognition and management of adverse drug reactions. The six hospitals serving the study area were visited monthly to check and collect inpatient forms, and to follow-up on any queries from forms collected in previous visits. When a suspected drug related adverse event was reported to study staff, the case was investigated by the local safety monitor who visited the child’s home to examine the child and interview the parents, met with health staff and checked health centre records, and completed an adverse event form and report. Severity was graded as mild (causing no or minimal interference with usual activities), moderate (symptoms interfering with usual activities), severe (symptoms preventing usual activities) or serious (potentially life-threatening). An independent panel reviewed severe and serious suspected adverse drug reactions and data from all inpatient records for children admitted to hospital for at least 24 hours within one month of SMC administration to evaluate severity and possible relationship (most probably, probably, possibly, unlikely, not related, insufficient to assess) with study drugs. Photographs of cases of severe skin rash were reviewed independently by two consultant dermatologists. Laboratories were visited to assess capacity for diagnostic investigations. Blood cell counts and liver function tests were performed in the three regional laboratories.

To obtain more details about the cases of vomiting, children who presented with vomiting to one 24 health posts within one week of the October 2010 SMC round were visited at home by project staff to ask parents or carers about the child's symptoms.

### Data analysis

Estimates of coverage of SMC doses and 95% confidence intervals were made using a ratio estimator, with each observation weighted by the inverse of the sampling fraction for the health post. Mortality rate ratios were estimated from DSS data on number of events and population at risk, using Poisson regression with a random effect. Analysis was done using Stata version 11 (Statacorp, College Station, Texas). A confidence interval for the ratio of the number of adverse events in two successive months, n_1_/n_2_, (n_1_ in the first month and n_2_ in the second month) was calculated as cL/(1-cL) and cU/(1-cU) where cL and cU are the lower and upper confidence limits on the binomial proportion n_1_/(n_1_+n_2_) [7]. A 95% confidence interval for the rate of adverse events was calculated by Wilson’s method [8].

### Analysis of inpatient and outpatient records

Inpatient records were entered by experienced data entry clerks who visited each hospital and entered the data onto a laptop in the hospital. Double data entry was not feasible, data were single-entered into an Access database, consistency checks were run as data were entered, any queries resolved with hospital staff, and further checks were run after data entry was completed. The primary diagnosis (the main reason for hospitalization) and secondary diagnoses (associated conditions needing treatment) were coded using a coding system that had been developed for use in the Niakhar Demographic Surveillance System. Coding was done independently by two physicians and discrepancies were resolved after discussion. Inpatient records of patients with a primary or secondary diagnostic code of jaundice, hepatitis, abdominal pain, isolated vomiting, diarrhoea, skin disease (dermatitis, rash, erythematous rash, macular rash, papular rash, maculo-papular rash, pruritic rash, pustular rash, vesicular rash), adverse drug reaction, food poisoning, or digestive disorder (nausea, vomiting, diarrhoea), and patients with a low white blood cell count, who had been admitted within 3 months from the date of the start of SMC administration, and who were residing in the study area, were extracted and classified as resident in or outside SMC administration areas on the basis of the village of residence recorded in the admission book. An attempt was made to link these records to the SMC administration records for the same village based on the name of the child and mother, and the child’s age. Where linkage was not possible it was assumed that SMC had been received on the date of the most recent round of SMC delivery in that village. The records were reviewed to assess their possible association with administration of SMC drugs. Outpatient records for children under 10 years of age seen at health posts from March to December 2008 were entered from health facility registers into an Access database, data were single entered, and completeness of data entry was checked by comparing the number entered with an independent tally of the number from the registers. In 2009 and 2010, outpatient attendances suspected to be related to drug intake were documented using pharmacovigilance forms. These forms were collected from all health facilities about 2 weeks after each round of SMC administration, and entered into a database (S1 Text).

### Ethics

A series of meetings were held with the local government authorities and district health staff to explain the aims and activities of the project and to seek approval from community leaders. On the first occasion when the intervention drugs were delivered through house to house visits, verbal consent to participate in the SMC programme was sought from the mother or carer of each eligible child by the CHW after explaining the programme using a standard script translated into the appropriate local language (Wolof or Serer). The Ethics committees which reviewed the protocol, approved the use of documented oral consent. The information sheet mentioned the aims of the project and the potential side effects of the study drugs. Verbal consent or refusal was recorded in a register by the CHW. Consent was sought separately for participation in demographic surveillance. The protocol was approved by the Ethics Committee of the London School of Hygiene&Tropical Medicine and by the Conseil national de recherche en santé (CNRS) in Senegal.

## Results

### SMC delivery and coverage

SMC was administered to about 14,000 children aged 3–59 months at the time of the first treatment in 2008, to about 90,000 children under 10 years of age in 2009 and to about 155,000 children under 10 years of age in 2010. The study profile is shown in Fig 2. The number of children who received the first daily dose of SMC treatment each year, documented in registers, is shown in Table 2. High coverage of three courses of treatment was achieved.

**Table 2:** No. of children who received the first daily dose of SMC treatment*.

	2008(3–59 months)	2009 (3–120 months)	2010 (3–120 months)	Total
Total	42,278	265,846	468,067	776,191

* These numbers exclude a small percentage of children (2.1% in 2008, 1.7% in 2009 and 0.68% in 2010) who refused treatment, spat it out or immediately vomited the first daily dose supervised by the CHW. Estimated coverage of three courses of treatment, determined when the resident population was surveyed at the end of the transmission season, was 92% (95%CI 90%, 95%) in 2008, 90% (88%,92%) in 2009 and 90% (95%CI 82%,97%) in 2010.

### Safety of SMC

#### Severe and serious adverse events

Five serious or severe adverse events were reported, including three which were considered to be possibly related to the intervention. These were an acute diarrhoeal illness in a 9-year old boy who died one week after the start of his first SMC course; rash and facial oedema in a 9-year-old boy which developed 2 days after the start of the first SMC course; and jaundice in a 5-year old boy which developed 2 days after the start of his second SMC course of treatment (signs of jaundice (yellowing of eyes and finger nails) were reported but liver function tests were not performed). An extra-pyramidal syndrome was diagnosed in an 8-year-old girl two days after the start of her first course of SMC treatment, and was probably related to SMC. A skin rash that developed 2 weeks after the start of the first course of SMC treatment in a boy aged 17 months, detected through active surveillance when the child was visited at home to administer the second round of SMC, was not thought to be related to SMC drugs. These children did not receive SMC again. The review panel considered that only the extra-pyramidal syndrome was likely to be related to study drugs. Photographs of the 17-month-old boy with skin rash were examined independently by two dermatologists who considered that its appearance was typical of the rash caused by staphylococcal infection. The case of jaundice might have been related to study drugs but the child had also taken other medicines including paracetamol, that might have caused liver injury. No serious adverse events attributable to SMC were reported after giving 776,191 documented treatments, giving an upper limit of the 95% confidence interval for the rate of serious events of 1 in 202,000. The incidence of extra-pyramidal syndrome was 1 in 776,191, with an upper 95% confidence limit of 1 in 137,000.

#### Deaths

The mortality rate from all causes among children eligible to receive SMC (i.e. aged 3–59 months in September 2008 or aged 3–119 months in September 2009 or September 2010), within one month of the date of an SMC round, detected through the DSS system, was similar in areas where SMC was delivered, and in non-SMC areas (mortality rate ratio SMC:non-SMC 0.93 [95%CI 0.69,1.25]). Verbal autopsies were conducted in the areas served by 12 of the 54 health posts, (2 of these implemented SMC in 2008, 6 in 2009 and 10 in 2010). Causes of death as determined by verbal autopsy are listed in Table 3.

**Table 3:** Causes of death determined by verbal autopsy among children eligible for SMC (i.e. aged 3–59 months in 2008 and aged 3–119 months in 2009 and 2010) in the areas served by 12 health post.

	2008		2009		2010	
	non-SMC	SMC	non-SMC	SMC	non-SMC	SMC
No. of health posts with VA investigation:	10	2	6	6	2	10
**Causes of death:**						
Malaria	14	2	3	1	0	1
Diarrhoea	7	6	0	6	0	1
Pneumonia	2	0	1	1	0	0
Septicaemia	0	1	0	0	0	0
Malnutrition	0	0	1	0	0	0
Severe abdominal illness	0	0	1	0	0	0
Renal infection	0	0	1	0	0	0
Severe anaemia	0	0	0	1	0	0
Congenital abnormalities	0	0	0	0	0	2
Snake bite	0	0	0	0	0	1
Lung abcess	0	0	0	0	0	1
Measles	0	0	0	0	0	1
Sickle cell disease	0	0	0	0	0	1
Other causes	7	0	0	0	0	3
TOTAL	30	9	7	9	0	11

#### Hospital admissions

In 2008, there were 3676 documented hospital admissions of children under five years of age of at least 24 hours duration in the six surveillance hospitals; 251 of these came from the study area and ninety-two were admitted in the period from the day of the start of SMC cycle 1 in September to one month after the last day of SMC administration in cycle 3 in November, eight from areas (9 health posts) where SMC was delivered in 2008 and 82 inpatients from areas (45 health posts) where SMC was not delivered in 2008 (S2 Fig). A total of 8 children were admitted to hospital during the period of SMC administration from areas where SMC was delivered (2 with diarrhoea, 1 with vomiting, 1 with ascites, 1 with malaria, 2 with pneumonia and 1 with convulsions).

In 2009, there were 4887 admissions among children under 10 years of age in the six surveillance hospitals, 305 from the study area, and 110 of these admitted during the period from the start of SMC administration in September up to one month after the last SMC administration, 52 from areas where SMC was being delivered and 58 from areas where SMC was not being delivered (S1 Fig). There were 3 admissions with jaundice or hepatitis as the primary or secondary diagnosis from the area where SMC was being delivered during the administration period—a newborn girl (age 4 days) with jaundice probably due to neonatal infection, a 12- month- old boy hospitalized for 10 days with a diagnosis of ascites, suggesting a chronic illness unlikely to be related to drug intake, and a girl aged 96 months admitted with a diagnosis of hepatitis without fever treated previously with a diuretic (furosemide), antihelmintic (albendazole) and iron.

In 2010, there were 5914 admissions among children under 10 years of age in the six surveillance hospitals, 446 of these came from the study area and of these 149 were admitted in the period from the day of the start of the first SMC cycle in September to one month after the last day of SMC administration November. Of these 149 patients, 136 came from areas (45 health posts) where SMC was delivered in 2010 and 13 from areas (9 health posts) where SMC was not delivered in 2010. No cases of hepatitis, jaundice or cutaneous eruption were recorded among the 136 children from SMC areas (S1 Fig).

#### Reports of mild and moderate adverse events

**Fig 3:**
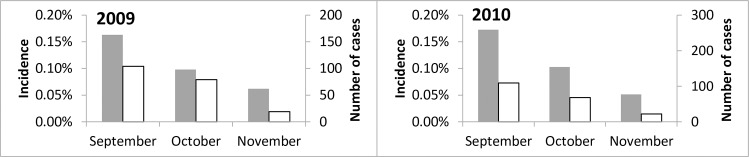
Adverse drug reactions notified by health facilities within 10 days of SMC administration. The incidence as the percentage of children who were treated is indicated on the left hand axis) and number of cases on the right hand axis). In 2009, the total number of adverse event reports was 33% (95% CI 19%,45%) lower in October than in September and 69% (95% CI 61%,76%) lower in November than in September. In 2010 the number of adverse events was 35% (95% CI 25%,44%) lower in October than in September, and in 70% (95% CI 64%,75%) lower in November.

**Fig 4:**
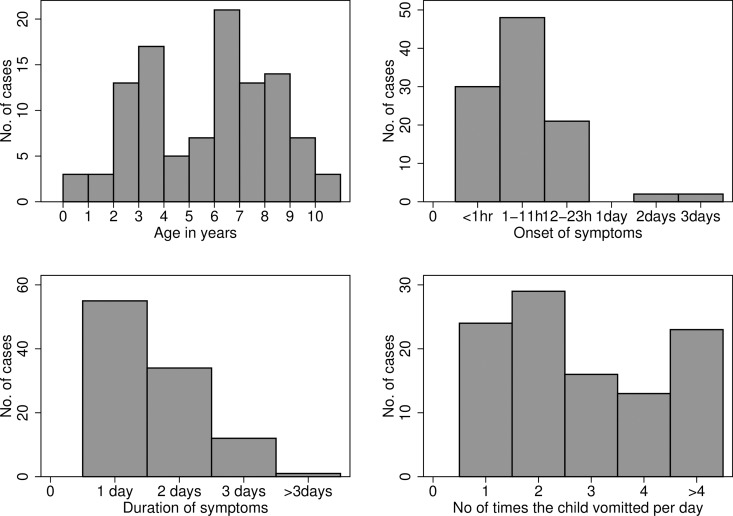
Age distribution of cases (upper left); time from the first SMC dose to onset of symptoms (upper right); duration of symptoms (lower left) and frequency of vomiting (lower right) in 108 children who presented at the clinic with symptoms of vomiting within 1 week of SMC administration.

In 2010, there were 924 reports of adverse drug reactions from the nurses of 45 health posts that delivered SMC, a rate per treatment of 0.14% (Table 4). Vomiting was the most commonly reported symptom. Incidence of adverse events decreased progressively in each successive month (Fig 3). Similar results were obtained in 2009 (S1 Table). One hundred and thirteen children who had presented within one week of SMC with vomiting were followed up in October 2010. Five children whose vomiting had started prior to SMC administration were excluded. The median age of the subjects was six years (52 boys and 56 girls), the median time of onset of symptoms was four hours after the first dose (Fig 4) and the mean duration of symptoms was 1.6 days. The age distribution was consistent with a dose-related effect, with peaks at two years of age (when the amodiaquine dose increased from 1/2 to 1 tablet) and at 6 years (when the dose rose from 1 to 1.5 tablets).

**Table 4:** Incidence of mild adverse reactions to SMC reported to health posts in 2010.

	Sep	Oct	Nov	Total
No. of children treated	154,013	157,602	159,667	471282
No. of children with reported adverse reaction	368	222	99	689
% children with reported adverse reaction	(0.2%)	(0.1%)	(0.06%)	(0.14%)
Total number of reported adverse reactions	474	307	143	924
Number reporting each type of symptom:				
Abdominal pain or vomiting	259	154	77	490
Fever	89	44	32	165
Headache	38	37	19	94
Diarrhoea	46	35	9	90
Itching/Rash	25	19	3	47
Drowsiness	6	8	2	16
Conjunctivitis	4	3	1	8
Oedema	3	2	0	5
Jaundice	0	2	0	2

## Discussion

From 2008 to 2010, about 780,000 treatment courses of SPAQ were administered to children living in three districts in Senegal, delivered by CHWs coordinated by district health staff. No serious adverse events definitively attributable to the intervention were detected, despite a high level of surveillance which included active follow-up of children as well as enhanced passive detection through health facilities by health staff who had been trained to recognise and document adverse reactions to SMC drugs and who were visited frequently by project staff.

Severe cutaneous reactions have been observed in adult travellers using SP weekly for prophylaxis [9]. Both SP and AQ have been associated with liver toxicity [10] and with agranulocytosis (a peripheral neutrophil count <0.5x10^9^ cells/L [10,11]) when used for prophylaxis in adult travellers from the US and Europe. Amodiaquine has been associated with serious and, in some cases, fatal bone marrow toxicity in adult European travellers when used as prophylaxis (a weekly dose of 400mg amodiaquine base). Agranulocytosis developed 5 to 14 weeks after the start of prophylaxis and was associated with hepatitis in some cases [9]. However the risk of these adverse reactions from routine use in African populations appears to be very low. There has been no increase in incidence of Stevens Johnson syndrome reported since SP became widely used for IPT in pregnancy. A study in Malawi showed that the incidence of severe skin reactions in children from use of SP for malaria treatment is very low, only 2 events were recorded in over 300,000 treatments [12]. A study of the safety of SP used for IPTi, through passive follow-up of 217,000 SP treatments and active follow up of 24,000 children treated with SP, found no serious adverse reactions [13] and an Institute of Medicine review [14] looked specifically at the safety of SP for IPTi and concluded that it was well tolerated. Our surveillance did not detect any cases of severe cutaneous reactions associated with SMC drugs. WHO commissioned a review of the safety of amodiaquine (AQ) in 2002 [15] including 270 prospective treatment and prophylaxis studies, and 73 retrospective studies. This review concluded that therapeutic treatment with AQ for malaria was well tolerated and AQ in combination with artesunate is now used widely as first line treatment for uncomplicated malaria.

In our study, the occurrence of hepatic disease in children was uncommon. 48 cases were detected, only two of whom had received SMC and could potentially be associated with drug intake. No cases of agranulocytosis were reported but this condition is not easily diagnosed in our setting. One child developed an extra-pyramidal syndrome after SMC treatment which was probably associated with amodiaquine intake. A review of 49 cases of extra-pyramidal syndrome associated with amodiaquine-artesunate treatment in Vigibase, the database of Individual Case Safety Reports maintained by WHO, suggests that there is an association with amodiaquine at recommended dosages [16]. Extra-pyramidal syndrome is an unpleasant but readily treatable condition. The incidence appears to be low but as it has not until recently been widely recognized as a potential side effect of amodiaquine treatment it may have been under-reported.

Vomiting was the most common side effect of SMC. The age pattern of vomiting in children who received SMC is consistent with a dose-related risk of vomiting, as has been reported in other studies of SMC in African children [17]. The overall rate of outpatient attendance with SMC-related vomiting was low. It is not clear why some children are more likely to experience vomiting than others. Variants in the CYP2C8 gene are associated with a reduced rate of metabolism of amodiaquine to its active antimalarial metabolite, N-desethylamodiaquine [18]. Therefore, people with these gene variants who eliminate amodiaquine more slowly than normal, especially homozygotes, may be at increased risk of adverse events related to amodiaquine. Parikh *et al*. [19], in a study of patients treated with amodiaquine-artsunate, found that heterozygotes and homozygotes for the CYP2C8*2 genotype were more likely to report abdominal pain compared to those with the wild-type, but there was no association with vomiting or other adverse events, and no evidence that treatment efficacy was impaired. The CYP2C8*3 variant, associated with more marked reduction in AQ metabolism, is uncommon in Africa [20]. The frequency of the CYP2C8*2 allele has been estimated to be 0.115 in Burkina faso [19]) and 0.168 and 0.179 in Ghana [21, 22,23]), with homozygote frequencies of 1%-3%.

In 2008 and 2009, 200 mg AQ tablets were used and 153mg breakable dispersible AQ tablets with sweetener in 2010. The use of the drug in liquid form may be better tolerated by young children and may permit more accurate dosing by age but may not be practical for community programmes where doses are left with the family to administer. Dosing by age used in this study followed the dosage scheme used for SPAQ when it was an interim first line treatment in Senegal. A slightly different dosage scheme by age is now recommended by WHO, with half tablets of SP (500/25 mg) and AQ (153mg) under 1 year of age and whole tablets for children aged 1–4 years [2].

The treatments were well tolerated. In 2009, mild adverse reactions were reported by only 530 children (0.2% of treatment courses), with vomiting and diarrhoea being the most commonly reported symptoms. Most reports were made after the September course of treatment with successively fewer complaints after the October and November courses. This trend, which has also been seen in clinical trials, probably reflects tolerance to side-effects after repeated doses, but may to some extent also reflect a tendency for mothers to be less likely to bring their child to the clinic as they became reassured of the safety of the treatments.

It is possible that some severe adverse drug reactions were not detected by our surveillance system. Agranulocytosis cases may have occurred but would have been difficult to diagnose in our setting. Guidelines for health staff could be improved by including severe sore throat with fever as a sign that could potentially be associated with agranulocytosis and should be investigated. For certain other conditions, including jaundice, many people prefer to consult a traditional healer, at least in the first instance. In our study area, traditional healers belong to one of six organisations and could potentially be included in a surveillance system. However, it is likely that more severe cases would be treated in health facilities. Although children may die at home without being seen by health staff, we have shown that there was no increase in child deaths associated with the intervention detected through the DSS system. This system may not have captured all deaths, but in twelve health posts, where village reporters were recruited to record all deaths in the village, investigation of cause of death using verbal autopsies did not suggest that any deaths were related to SMC drugs. A further strength of our study is that, since the intervention comprises three rounds of SMC a month apart, active follow-up allowed surveillance for any severe adverse reactions after the first and second course. Investigation of possible causes of adverse events was limited by laboratory capacity in the health facilities but basic haematological and biochemical tests could be done in the hospitals. Nevertheless it is reassuring that assessment based on clinical symptoms found no evidence of an increase in hospital admissions associated with adverse drug reactions to SMC.

We did not find evidence that SMC reduced mortality but the mortality rates were very low in the study area and the confidence interval for the effect of SMC did not rule out a substantial benefit. Child survival in this area has recently improved, associated with a dramatic reduction in the incidence of malaria [24]. The low malaria transmission in the study area meant it was highly unlikely that there could have been an increase in deaths due to adverse drug reactions that was obscured by a reduction in deaths from malaria.

Where SMC is implemented, effective pharmacovigilance should be maintained but this may require substantial investment in training, coordination and logistics, as African pharmacovigilance systems are known to be weak [25]. In Senegal, although a national pharmacovigilance plan has been developed and training workshops organized in all the health districts [26], many of the country’s health districts do not submit reports. African countries as a whole contribute only 0.3% of the ICSRs (Individual Case Safety Reports) in the WHO database [27]. Re-design of reporting forms may be needed to ensure all relevant information is captured, and improved systems for collection and processing of pharmacovigilance reports need to be established. In our study, the system was strengthened by ensuring health staff had forms and guidelines, text messages were sent to nurses to remind them about pharmacovigilance, forms were collected regularly and results fed back to health staff. The first dose of each treatment course was observed or given by the CHW and documented in the register and on the child’s record on the mother’s DSS card. Families were encouraged to report any side effects and health staff were trained to recognize symptoms and report promptly. The Global Fund encourages funding requests to improve pharmacovigilance in countries that lack pharmacovigilance capacity [28]. Provision for strengthening national pharmacovigilance systems should be included in plans for SMC implementation.

This study has shown that high coverage can be achieved when the intervention is delivered by district health teams and that SMC with SPAQ is well-tolerated when delivered on a large scale. These findings should support the introduction of the intervention in areas where seasonal malaria continues to cause severe illness and mortality among children. SMC is recommended for children under five years of age, but in some areas the age distribution of disease burden may justify extending this age limit, as has been done in Senegal. Our results show that SMC is well tolerated in children under five years of age and in older children.

## Supporting Information

S1 Fig Information sheet for health workers. (DOCX)

S2 Fig Hospital admissions for 2008–2010. (DOCX)

S1 Table Mild adverse reactions to SMC reported to health posts in 2009. (DOCX)

S1 Text Methods for review of inpatient records. (DOCX)
