# Correction: Safety of Seasonal Malaria Chemoprevention (SMC) with Sulfadoxine-Pyrimethamine plus Amodiaquine when Delivered to Children under 10 Years of Age by District Health Services in Senegal: Results from a Stepped-Wedge Cluster Randomized Trial

**DOI:** 10.1371/journal.pone.0168421

**Published:** 2016-12-08

**Authors:** J. L. NDiaye, B. Cissé, E. H. Ba, J. F. Gomis, C. T. Ndour, J. F. Molez, F. B. Fall, C. Sokhna, B. Faye, E. Kouevijdin, F. K. Niane, M. Cairns, J. F. Trape, C. Rogier, O. Gaye, B. M. Greenwood, P. J. M. Milligan

The following information is missing from the Funding section: MC received support through a Population Health Scientist Fellowship jointly funded by the UK Medical Research Council (MRC) and the UK Department for International Development (DFID). JLN is supported by a Wellcome Trust Intermediate Fellowship in Public Health and Tropical Medicine.
