# Supplementary material for: Safety of Seasonal Malaria Chemoprevention (SMC) with Sulfadoxine-Pyrimethamine plus Amodiaquine when Delivered to Children under 10 Years of Age by District Health Services in Senegal: Results from a Stepped-Wedge Cluster Randomized Trial
Source: PLoS One. 2016 Oct 20;11(10):e0162563. doi: 10.1371/journal.pone.0162563 (PMC5072628; doi:10.1371/journal.pone.0162563)
Supplement: S1 Fig — (DOCX) [file pone.0162563.s001.docx]

S1 Fig Information sheet for health workers.
