# Supplementary material for: Safety of Seasonal Malaria Chemoprevention (SMC) with Sulfadoxine-Pyrimethamine plus Amodiaquine when Delivered to Children under 10 Years of Age by District Health Services in Senegal: Results from a Stepped-Wedge Cluster Randomized Trial
Source: PLoS One. 2016 Oct 20;11(10):e0162563. doi: 10.1371/journal.pone.0162563 (PMC5072628; doi:10.1371/journal.pone.0162563)
Supplement: S2 Fig — (DOCX) [file pone.0162563.s002.docx]

S2 Fig. Hospital admissions for 2008-2010

2008:

3676 inpatient records <5yrs*

251 in the DSS area

3096 live outside the DSS area

329 area of residence unknown

17 age missing

67 age out of range

168 cases 0-5yrs in the DSS area

76 outside the 3-month period of SMC administration

92 inpatients 0-5yrs admitted in the period from the day of the start of SMC round 1 in September to one month after the last day of SMC administration in round 3 (November):

8 inpatients from areas (9 health posts) where SMC was delivered in 2008

84 inpatients from areas (45 health posts) where SMC was not delivered in 2008

2009:

4887 inpatient records <10yrs*

305 in the DSS area

3972 live outside the DSS area

610 area of residence unknown

27 age missing

278 cases 0-10yrs in the DSS area

168 outside the 3-month period of SMC administration

110 inpatients 0-10yrs admitted in the period from the day of the start of SMC round 1 in September to one month after the last day of SMC administration in round 3 (November):

52 inpatients from areas (27 health posts) where SMC was delivered in 2009

58 inpatients from areas (27 health posts) where SMC was not delivered in 2009

2010:

5914 inpatient records <10yrs*

446 in the DSS area

4900 live outside the DSS area

568 area of residence unknown

13 age missing

433 cases 0-10yrs in the DSS area

284 outside the 3-month period of SMC administration

149 inpatients 0-10yrs admitted in the period from the day of the start of SMC round 1 in September to one month after the last day of SMC administration in round 3 (November):

136 inpatients from areas (45 health posts) where SMC was delivered in 2010

13 inpatients from areas (9 health posts) where SMC was not delivered in 2010
