# Supplementary material for: Safety of Seasonal Malaria Chemoprevention (SMC) with Sulfadoxine-Pyrimethamine plus Amodiaquine when Delivered to Children under 10 Years of Age by District Health Services in Senegal: Results from a Stepped-Wedge Cluster Randomized Trial
Source: PLoS One. 2016 Oct 20;11(10):e0162563. doi: 10.1371/journal.pone.0162563 (PMC5072628; doi:10.1371/journal.pone.0162563)
Supplement: S1 Table — (DOCX) [file pone.0162563.s003.docx]

S1 Table. Mild adverse reactions to SMC reported to health posts in 2009.

|  | Sep | Oct | Nov | Total |
| --- | --- | --- | --- | --- |
| No. of children treated | 89347 | 89405 | 91694 | 270446 |
| No. of children with reported adverse reaction | 268 | 180 | 82 | 530 |
| % children with reported adverse reaction | 0.30% | 0.20% | 0.09% | 0.20% |
|  |  |  |  |  |
| Abdominal pain or vomiting | 163 (61%) | 98 (54%) | 62 (76%) | 323 (61%) |
| Diarrhoea | 29 (11%) | 29 (16%) | 2 (2.4%) | 60 (11%) |
| Drowsiness | 10 (3.7%) | 7 (3.9%) | 1 (1.2%) | 18 (3.4%) |
| Fever | 19 (7.1%) | 27 (15%) | 5 (6.1%) | 51 (9.6%) |
| Headache | 20 (7.5%) | 7 (3.9%) | 11 (13%) | 38 (7.2%) |
| Itching | 24 (9.0%) | 9 (5.0%) | 0 (0.0%) | 33 (6.2%) |
| Rash | 2 (0.7%) | 0 (0.0%) | 0 (0.0%) | 2 (0.4%) |
