# Supplementary material for: Safety of Seasonal Malaria Chemoprevention (SMC) with Sulfadoxine-Pyrimethamine plus Amodiaquine when Delivered to Children under 10 Years of Age by District Health Services in Senegal: Results from a Stepped-Wedge Cluster Randomized Trial
Source: PLoS One. 2016 Oct 20;11(10):e0162563. doi: 10.1371/journal.pone.0162563 (PMC5072628; doi:10.1371/journal.pone.0162563)
Supplement: S1 Text — (DOCX) [file pone.0162563.s004.docx]

S1 Text. Methods for review of inpatient records.

Inpatient records were entered by experienced data entry clerks who visited each hospital and entered the data onto a laptop in the hospital. Data were single-entered into an Access database, consistency checks were run as data were entered and after data entry was competed. The primary diagnosis (the main reason for hospitalization) and secondary diagnoses (associated conditions needing treatment) were coded using a coding system that had been developed for use in the Niakhar Demographic Surveillance System. Coding was done independently by two physicians and discrepancies were resolved after discussion. Inpatient records of patients with a primary or secondary diagnostic code of jaundice, hepatitis, abdominal pain, isolated vomiting, diarrhoea, allergic or non-allergic skin disease, adverse drug reaction, food poisoning, or digestive disorder (nausea, vomiting, diarrhoea), who had been admitted within 3 months from the date of the start of SMC administration, and who were residing in the study area, were extracted and classified as resident in or outside SMC administration areas on the basis of the village of residence recorded in the admission book. An attempt was made to link these records to the SMC administration records for the same village based on the name of the child and mother, and the child’s age. Where linkage was not possible it was assumed that SMC had been received on the date of the most recent round of SMC delivery in that village. The records were reviewed to assess their possible association with administration of SMC drugs. Outpatient records for children under 10 years of age seen at health posts from March to December 2008 were entered from health facility registers into an Access database, data were single entered, and completeness of data entry was checked by comparing the number entered with an independent tally of the number from the registers. The number of malaria cases by month and age group were compared with the number tallied by the health post nurse and with the number tallied by a project field supervisor. In 2009 and 2010, outpatient attendances suspected to be related to drug intake were documented using pharmacovigilance forms. These forms were collected from all health facilities about 2 weeks after each round of SMC administration, and entered into a database.
